# Research on Slurry Flowability and Mechanical Properties of Cemented Paste Backfill: Effects of Cement-to-Tailings Mass Ratio and Mass Concentration

**DOI:** 10.3390/ma17102222

**Published:** 2024-05-08

**Authors:** Yan Li, Jianxin Fu, Jiguang Yang, Jie Wang

**Affiliations:** 1 School of Civil and Resources Engineering, University of Science and Technology Beijing, Beijing 100083, China; liyan6816@163.com (Y.L.); goodyangjiguang@163.com (J.Y.); 2 Key Laboratory of High-Efficient Mining and Safety of Metal Mines, Ministry of Education, University of Science and Technology Beijing, Beijing 100083, China; 3 Backfill Engineering Laboratory, Shandong Gold Mining Technology Co., Ltd., Laizhou 261441, China

**Keywords:** cemented tailings backfill, rheological properties, loop test, pipeline transportation, unconfined compressive strength

## Abstract

The flowability and mechanical properties are increasingly crucial in the filling process of deep metal mines with mining depths exceeding 1000 m. The rheological properties of filling slurry in the pipeline were analyzed through rheological tests, L-tube self-flow tests, and semi-industrial loop tests. The results revealed that with an increase in the cement-to-tailings mass ratio (c/t ratio) and mass concentration, the slurry exhibited a higher flow resistance and decreased stowing gradient. During slurry transportation, the pressure loss in the straight pipe was positively correlated with the slurry flow rate, c/t ratio, and mass concentration. A uniaxial compressive strength (UCS) test was conducted to analyze the mechanical properties of the cemented paste backfill containing BMC (CCPB) in both standard and deep-underground curing environments. The UCS of the CCPB showed an increasing trend with the rise in curing age, mass concentration, and the c/t ratio. The comprehensive analysis concluded that when the c/t ratio is 1:4, and the mass concentration is approximately 74%, and parameters such as the slump, bleeding rate, and flowability of the filling slurry meet the criteria for conveying and goaf filling, resulting in a high-strength filling body.

## 1. Introduction

As mining operations extend to kilometer depths in various countries due to depleted shallow surface mineral resources [1,2]. Goaf filling has become an indispensable part of the mining process to effectively support the goaf, prevent ground subsidence, and reduce solid waste discharge [3,4,5]. Slurry segregation, stratification, and bleeding often occur during high-depth, long-distance filling processes, negatively affecting slurry transportation and the strength of cemented paste backfill (CPB) [6,7]. The total tailings paste filling technology is maturing [8,9,10]. The paste possesses stability, fluidity, and plasticity, with high anti-segregation properties, a dense structure, and high strength of the filling body [11,12,13]. The technology decreases cement usage, drastically reducing filling and mining expenses, and therefore, features the advantages of economy, safety, and environmental protection [14,15,16]. Paste in the pipeline in the form of a high-concentration structural flow medium exhibits a complex rheological behavior and flow mechanism, falling within the realm of multidisciplinary cross-discipline research [17,18]. Further research into the technological parameters of paste filling is urgently needed to achieve excellent transportation performance and mechanical properties of the paste slurry [19,20].

Total tailings paste pipeline transportation can be divided into the self-flow method, which relies on the slurry’s gravity, and the pumping method, which uses external force [21,22,23]. The total tailings paste slurry possesses a relatively large plastic viscosity, yield stress, and pipeline transportation resistance, normally adopted by pumping transportation [24,25,26]. However, as mining depths continue to increase, the advantages of self-flow transportation aided by gravitational systems are becoming increasingly significant [27,28]. Currently, the stowing gradient of total tailings paste is mainly investigated by implementing an indoor rheometer test, L-tube self-flow test, and semi-industrial loop test [29,30,31,32]. The L-tube self-flow test simulates the slurry flow properties and transportation resistance, facilitating the assessment of the slurry transportation range according to the stowing gradient, effectively addressing self-flow transportation challenges [33,34,35]. Compared with other experimental methods, the loop test provides a high degree of accuracy in the paste flow resistance measurement, thereby providing robust theoretical support for practical projects to ensure safe and reliable slurry transportation [36,37,38].

The rheological properties of a CPB slurry are essential parameters in the transportation and filling process [39,40]. Many scholars have conducted experimental studies on the rheological properties of filling slurries [41,42], especially on the mass concentrations of slurries, materials such as flocculants and water-reducing agents, tailings particle size distributions and properties, temperatures and PH values, and other external environments [43,44,45]. Jiang et al. [46] measured the yield stress using the vane method and concluded that the yield stress of a slurry decreases with an increasing salt content. Zhu et al. [47] studied the effect of the solution’s pH on the flocculation, rheological, and interfacial properties of slurries, and the yield stress of the slurry was significantly increased by more than 1000 times at pH 12. Ruan et al. [48] quantified the influence of flocculant addition and pH on the yield stress, indicating that it significantly affects the concentration and flow performance of the slurry. Wu et al. [49] established a yield stress prediction model by combining the true density of tailings, bulk density, and mass concentration, and concluded that the yield stress of a freshly mixed thickened tailings slurry is proportional to the true bulk density and mass concentration and inversely proportional to the bulk density.

Accordingly, this paper investigates the transportation characteristics and macromechanical properties of a slurry utilizing backfilling material C (BMC) as the cementitious material and cemented paste backfill containing BMC (CCPB). We conducted slump tests, rheology tests, L-tube self-flow tests, and semi-industrial loop tests for the filling slurry to analyze its flow performance from multiple perspectives. Additionally, uniaxial compressive strength (UCS) tests were conducted on CCPB to analyze the dynamic characteristics of the filling body, which were combined with the slurry transportation properties to summarize the most appropriate engineering parameters for this mine. This research provides fundamental data and theoretical guidance for high-depth and long-distance mine filling projects.

## 2. Materials and Methods

### 2.1. Experimental Materials

The filling aggregates, cementitious materials, and mixing water selected for this experiment were analyzed for their basic properties and conformed to the requirements of mine production.

#### 2.1.1. Filling Aggregate

**Figure 1:**
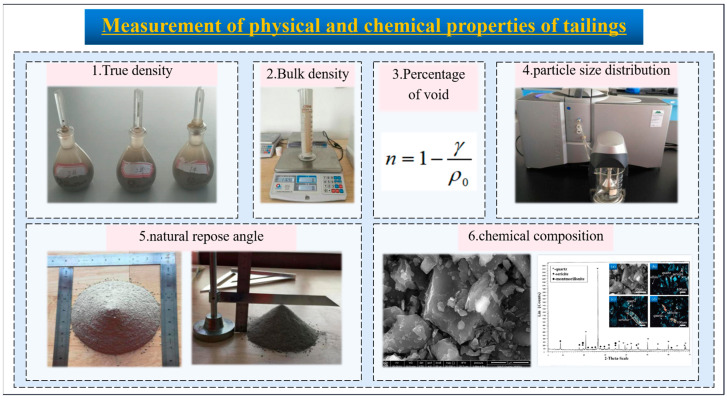
Measurement of physical and chemical properties of tailings.

**Figure 2:**
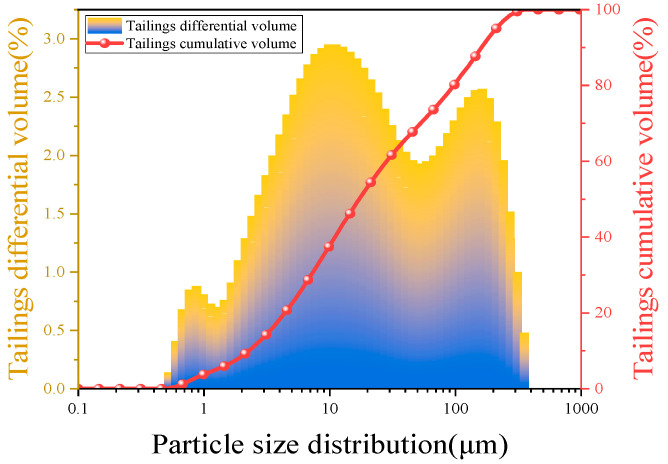
Particle size distribution curve of tailings.

In this experiment, tailings from mining enterprises in Shandong, China, were collected as filling aggregates. The physicochemical properties were tested as shown in Figure 1, and the physical parameters are presented in Table 1. Figure 2 illustrates the experimental data from the particle size distribution (PSD) test using a Malvern laser particle size analyzer. d_10_, d_60_, and d_90_ represent the diameter of the sieve through which particles with cumulative contents of 10%, 60%, and 90% can pass, respectively. d_10_ = 2.58 μm, d_60_ = 19.5 μm, and d_90_ = 183 μm. The tailings exhibited a highly variable grain size, lack of an intermediate portion of the grain size composition, and a high abundance of coarser and finer grains.

**Figure 3:**
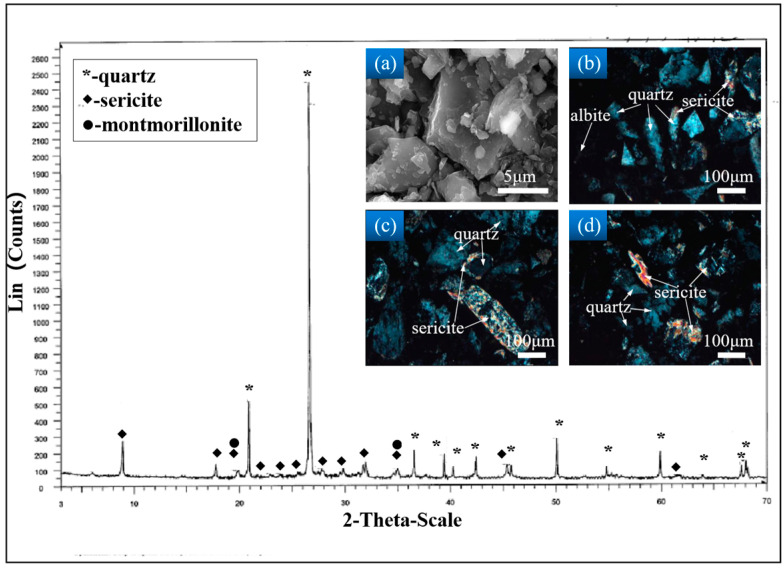
XRD phase analysis results of tailings: (**a**) 5 μm topography of tailings; (**b**) quartz is irregular granular output; (**c**) sericite and quartz are produced together; (**d**) sericite is flaky output.

**Figure 4:**
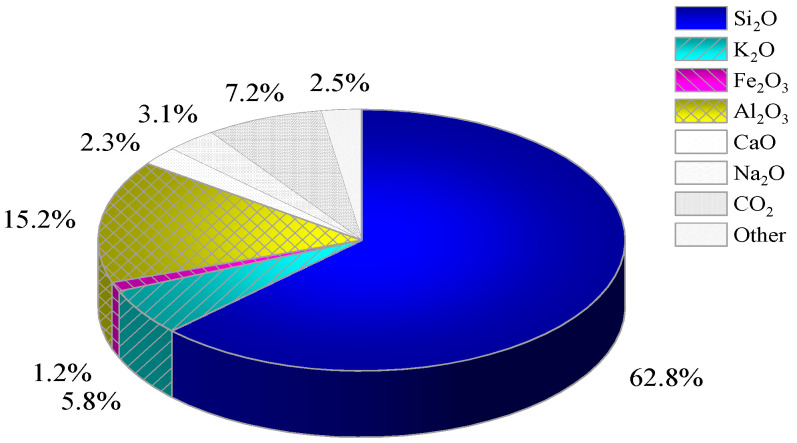
Chemical composition analysis of tailings.

Figure 3 illustrates the results of the physical phase analysis of the whole tailings. The physical phase composition of the tailings selected by the mine is mainly composed of quartz and mica, and the internal particles of the tailings can be seen in Figure 3a, which are of irregular particle sizes and a disordered arrangement. From Figure 3b–d, it can be seen that the quartz inside the tailings is mainly produced as irregular granular monomers, with a small amount of sericite being produced alongside it. Sericite is often presented as a flaky or scaly aggregate, which is hardly soluble in water and has a small impact on the fluidity of the filling slurry and the strength of the CCPB. Figure 4 presents the chemical composition analysis results of the tailings, showing that the contents of SiO_2_ and Al_2_O_3_ are relatively high.

#### 2.1.2. Cementitious Material

**Figure 5:**
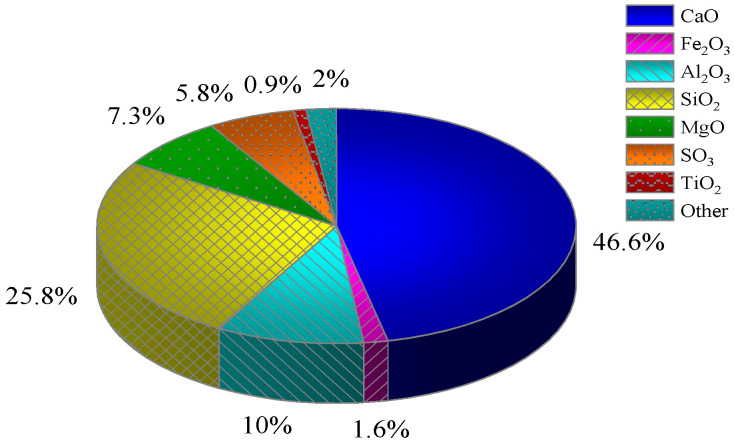
Chemical composition analysis of BMC.

The cementitious material is backfilling material C (BMC). BMC composed of slag, gypsum, and other substances is the special cementitious material for this mine; the main physical parameters are shown in Table 2, and the main chemical composition is shown in Figure 5.

#### 2.1.3. Mixing Water

The mixing water used in the experiment was mine production water. The pH of the production water was tested to be 7.2; the content of suspended solids was 120 mg/L; ammonia nitrogen, copper, lead, and zinc were not detected; and the concentration of chlorine ions was 15,171 mg/L. The production water from the mine was applicable for underground filling work.

### 2.2. Sample Preparation

In order to study the impact of different mass concentrations and cement-to-tailings mass ratios (c/t ratio) on the rheological and mechanical properties of the filling body, five groups of different c/t ratios, five groups of varying mass concentrations, and four groups of different curing ages were selected as the experimental variables. The filling body, utilizing BMC as the cementitious material, is abbreviated as CCPB. The mass concentration of the filling slurry ranged from 70% to 78%, the c/t ratio varied from 1:20 to 1:4, and the curing ages were 3 days, 7 days, 14 days, and 28 days. Before preparing the CCPB, lubricating oil was applied inside the mold to ease mold removal. According to the requirements of the mass concentration, the weighed tailings and cementitious materials were poured into the mixing container for dry mixing, and the cementitious sand mixer was used for powerful mixing after adding water. In accordance with the test program, the slurry was slowly infused into the 7.07 cm × 7.07 cm × 7.07 cm standard triple test mold. Each time after the slurry was added, the mold was struck with a small iron bar to avoid mixing air, then left to naturally settle, and the slurry was added on time to ensure that the size of the CCPB after curing was standardized. Due to the variety of slurry mass concentrations and c/t ratios, the specimen can be initially self-supporting after only 1–3 d. Then, it can be demolded and cured. The specimens were placed in a standard constant temperature and humidity curing chamber until they reached the specified ages, and the curing environment was further categorized into standard and deep-underground curing environments. The curing temperature of the standard curing environment was maintained at 20 °C, and the humidity was 90%. The deep-underground curing environment was close to the mine’s underground depth of 1000 m, and the curing temperature was maintained at 30 °C with a humidity of 90%.

### 2.3. Experimental Procedure

**Figure 6:**
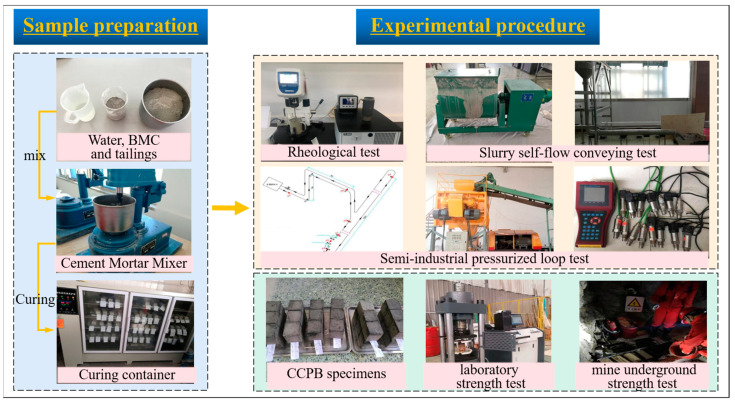
Flow chart of experiment.

On the one hand, the pipeline transportation resistance and stowing gradient of total tailings paste were investigated, and the experiments encompassed rheometer tests, L-tube self-flow tests, and semi-industrial loop tests. On the other hand, uniaxial compressive strength tests in different curing environments and a slump test for the slurry were conducted to measure the specimen consolidation characteristics and strength indexes. The overall experimental process is shown in Figure 6. 

#### 2.3.1. Filling Slurry Transportation Test

With the mining depth of the mine reaching 1000 m, in order to fully utilize the gravity advantage of the mine, the following filling slurry transportation experiments were carried out primarily to study the stowing gradient and flow resistance along the pipeline transportation, and then determine whether the self-flowing transportation could be applied successfully to the mine:

1. Rheological testing: The rheological characterization of total tailings paste was carried out with a variable shear method to obtain the rheological parameters of the total tailings paste. The CSR mode of the rheometer was selected for the test, and the maximum shear rate was 100 s^−1^. Considering the dynamic equilibrium state of the slurry during the test process, the test was set for a certain time (1200 s) of constant shear. The specific loading path was that the shear rate was incremented from 0 s^−1^ to 100 s^−1^, then decremented to 0 s^−1^ after the shear rate was steady at 100 s^−1^.
2. L-pipe self-flow test: A small-scale simulation test for L-type pipeline transportation was conducted according to a certain c/t ratio and mass concentration. The filling slurry was thoroughly mixed and poured into the upper hopper. Utilizing gravity, the slurry flowed downward through the L-type pipeline, which was connected to the hopper, and exited from the lower outlet. The vertical pipe height was h = 1.05 m; the horizontal pipe length was L = 2 m; the inner diameter of the pipeline was D = 0.1 m. By measuring the height of the stationary slurry column and other values, we could calculate the flow rate of the slurry transportation, the yield stress, the plastic viscosity coefficient, the shear stress of the wall of the pipe, the stowing gradient, and other related parameters.
3. Semi-industrial loop test: The semi-industrial loop system simulates the filling slurry pipeline arrangement of the mine. It tests the pipeline pressure loss and flow properties of different mass concentrations and c/t slurry ratios in the process of pipeline transportation in the case of continuous pumping. The pipeline pressure data were processed using the weighted arithmetic average method to obtain the average value of the pressure p*, and the pressure loss △p* of different pipeline sections was calculated accordingly.

#### 2.3.2. Mechanical Properties Test

Three test blocks were taken at each curing age to measure the UCS, and the value of the UCS was the average value of them. Before conducting the pressure test, the standard force measuring ring was used to adjust the press to ensure the accuracy of the test results. The different curing environments were divided into standard and simulated deep-underground curing environment UCS tests. By considering the strength index requirements for underground mining operations on the filling body, the optimal filling parameters of this mine were obtained through a comprehensive analysis.

## 3. Results

### 3.1. Experimental Results

Table 3 shows the rheological parameters obtained from the L-tube self-flow test. After obtaining the flow quantities, mass concentrations, and pipeline diameters of different slurries, the flow resistance and the stowing gradient for smooth pipeline transportation were calculated, as shown in Table 3. With an increase in the c/t ratio and mass concentration, the flow resistance of the slurry in the pipe increases and the stowing gradient decreases. As the mass concentration increases from 74% to 76%, the transportation resistance of the slurry increases sharply, so the concentration of the slurry should be less than 76%.

In the process of filling slurry transportation, the measurement of pipeline pressure loss is of great significance to the layout of the filling pipeline and equipment selection. Table 4 shows the semi-industrial loop test data under the condition of a c/t ratio of 1:4. The pipeline pressure loss values for 30° inclined and vertically downward slurry transportation are relatively small, and the pipeline pressure loss values for vertically upward slurry transportation are huge.

### 3.2. Analysis of the Slump and Bleeding Rate

#### 3.2.1. Standard Curing Environment

**Figure 7:**
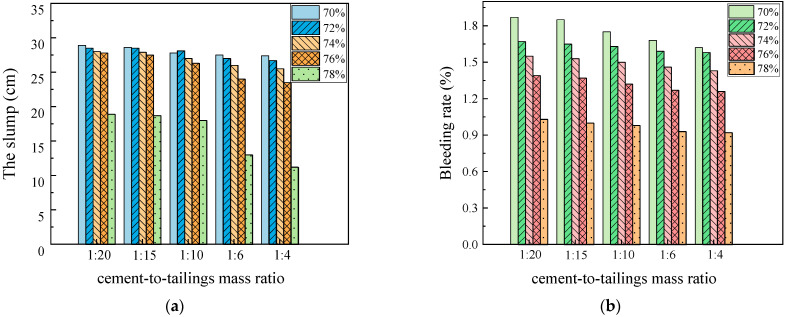
Experiments result of slump and bleeding rate experiments in standard laboratory curing environment. (**a**) the slump; (**b**) the bleeding rate.

The experimental results of each group in the standard curing environment are illustrated in Figure 7. As depicted in Figure 7, with an increase in the slurry c/t ratio and mass concentration, the slump and bleeding rate of the filling slurry showed a decreasing trend. From a comprehensive point of view, the slump and bleeding rate are insignificantly affected by the c/t ratio and are relatively remarkably affected by the mass concentration. Taking the c/t ratio 1:4 as an example, when the mass concentration ranges from 70% to 76%, there is little difference in the slump, and the slump reduction rate is 14.23%; when the mass concentration varies from 76% to 78%, there are excessive solid particles inside the slurry, and the lubricity and fluidity are inferior, so the slump reduction rate reaches 52.34%. According to the literature [50], slumps exceeding 25 cm indicate an excellent pipeline transportation performance; it can be judged that when the mass concentration is 78%, the filling slurry inside the pipeline is prone to plugging and other problems, which is not conducive to stope filling operations. There exists a negative correlation between the bleeding rate and mass concentration. As the mass concentration increases, the bleeding rate of the filling slurry shows a continuously decreasing trend, but the trend progressively shows a saturated state. The trend is that the amount of mixing water used in the slurry reduces with the growth in mass concentration. During the static settlement process of filling slurry, cementitious materials undergo hydration reaction to absorb free water [51,52]. Therefore, the reduced surplus water within the filling slurry inhibits water seepage, which is conducive to controlling water seepage and facilitating the rapid consolidation of the filling body. When the mass concentration is 70%, the bleeding rate is comparatively high, which will easily result in segregation, delamination, and water seepage in the filling slurry when filling processes occur at high depths and long distances, affecting the strength of the filling body and the overall filling condition. By comprehensively analyzing the relationship between the bleeding rate and the slump, it can be inferred that when the slurry mass concentration is 72%, 74%, and 76%, the slurry transportation performance is suitable for the deep-underground curing environment experiment.

#### 3.2.2. Deep-Underground Curing Environment

**Figure 8:**
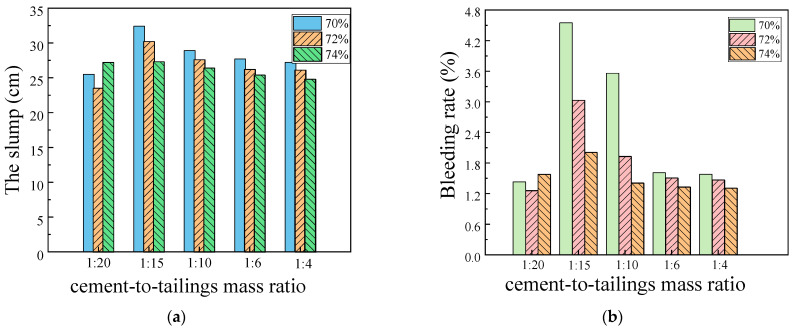
Experiments result of slump and bleeding rate experiments at deep-underground curing environment. (**a**) the slump; (**b**) the bleeding rate.

The slump and bleeding rate experiments were also carried out in the deep-underground curing environment. The variation characteristics of the former with different c/t ratios and mass concentrations are shown in Figure 8. It can be seen from Figure 8 that in the high temperature and high humidity environment, with the growth in the c/t ratio, the slump and bleeding rate present a trend of increasing first and then declining, and the slump and bleeding rate reach the peak value when the c/t ratio is 1:15. When the mass concentration of filling slurry is 72%, the bleeding rate fluctuates considerably, and as the c/t ratio rises from 1:20 to 1:4, the variation of the bleeding rate reaches 195.10%, −15.64%, −54.78%, and −1.86%. Especially when the c/t ratio is 1:15, its bleeding rate reaches 4.22%, which will lead to serious bleeding of fresh mortar, and the free water that segregates out will further affect the solidification and hardening of the slurry, which is unfavorable to the practical application of the project. With the mass concentration at 74%, as the c/t ratio increased from 1:20 to 1:4, the bleeding rate of the slurry was 1.26%, 3.03%, 1.93%, 1.51%, 1.47%, corresponding to slumps of 23.5 cm, 30.2 cm, 27.6 cm, 26.2 cm, and 26.1 cm, respectively. Considering the above experimental results, it was concluded that when the mass concentration of the filling slurry was about 74%, the indexes of the slump, the extension, and the bleeding rate of the filling slurry could meet the requirements of slurry transportation and stope filling.

### 3.3. Analysis of Slurry Flow Properties

#### 3.3.1. Rheological Test Results

**Figure 9:**
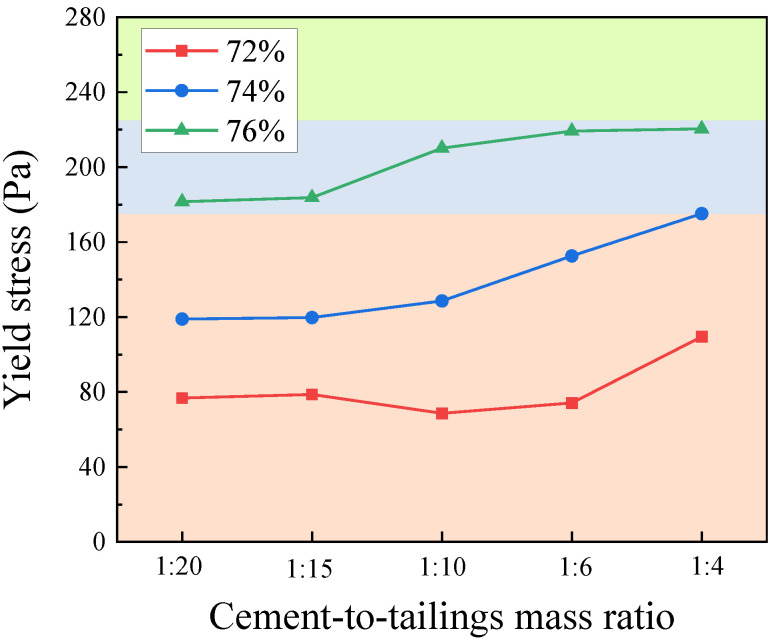
Yield stress with different mass concentrations and c/t ratios.

The yield stress of filling slurry with different mass concentrations varies with an increase in the c/t ratio, as depicted in Figure 9. Additionally, the yield stress is positively correlated with its mass concentration and c/t ratio. When the c/t ratio increases from 1:20 to 1:4, the yield stress increases by 42.62%, 47.35%, and 21.43%, respectively. When the mass concentration is 76% and the c/t ratio is 1:4, the yield stress reaches a maximum value of 220.4 Pa. The slope of the curve reflects the growth rate of yield stress within this range. As the mass concentration of the paste increases, the interval of the top growth rate of yield stress under different c/t ratios shifts forward. According to the rheological theory of paste, when the yield stress is 200 ± 25 Pa, the filling slurry can be regarded as a paste [53]. When the yield stress is beyond this range, the slurry mass concentration is too small or too large, which demands high-tech and economic support for pipeline transportation in the mine, so it is unsuitable for pipeline transportation. Figure 9’s blue area, representing the filling slurry in the paste state, is suitable for mining industry pipeline transportation. For example, when the c/t ratio is 1:4, the filling slurry with a mass concentration between 74% and 76% can be regarded as a paste.

#### 3.3.2. L-Tube Self-Flow Simulation Experiment Results

**Figure 10:**
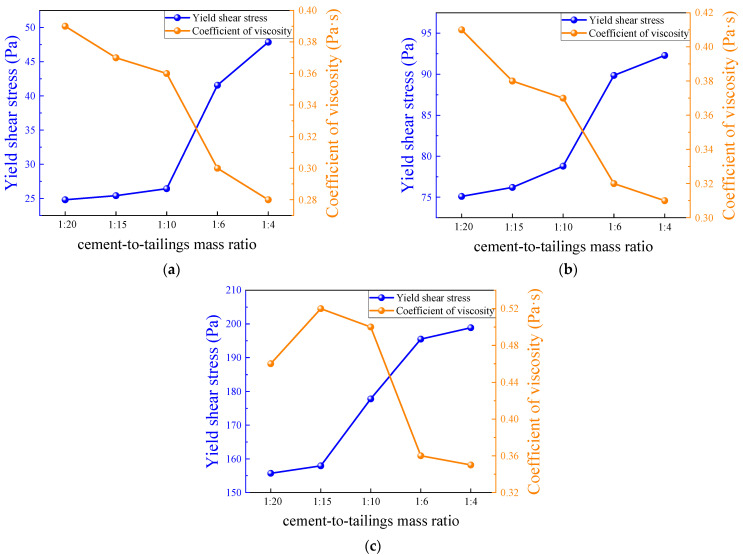
Yield shear stress and coefficient of viscosity for different c/t ratios: (**a**) 72%; (**b**) 74%; and (**c**) 76%.

The variation in the yield shear stress and coefficient of viscosity with the c/t ratio is illustrated in Figure 10, which reveals an upward trend in the yield shear stress as c/t ratio increases. On the contrary, the coefficient of viscosity exhibits an overall downward trend. The ability of the slurry to resist shear deformation in a static state is highly correlated with the c/t ratio and mass concentration. When the c/t ratio is 1:4, as the mass concentration of the slurry increases from 72% to 76%, its yield shear stress remarkably rises from 47.86 pa to 198.87 pa, marking an increment of 315.52%. When the slurry mass concentration is 76% and the c/t ratio is increased from 1:20 to 1:4, its yield shear stress shows an improvement of 27.73%, rising from 155.70 pa to 198.87 pa. In particular, when the c/t ratio increases from 1:10 to 1:6, the slope of the curve is the largest; the data exhibits the most pronounced increase and decrease trends; the rheological properties of the change are more remarkable; the yield shear stress increased by 57.28%, 14.05%, and 9.96%; and the coefficient of viscosity decreased by 16.67%, 13.51%, and 28.00%, respectively. When the c/t ratio increases to 1:4, the yield stress continues to grow and reaches the maximum value within the test group.

**Figure 11:**
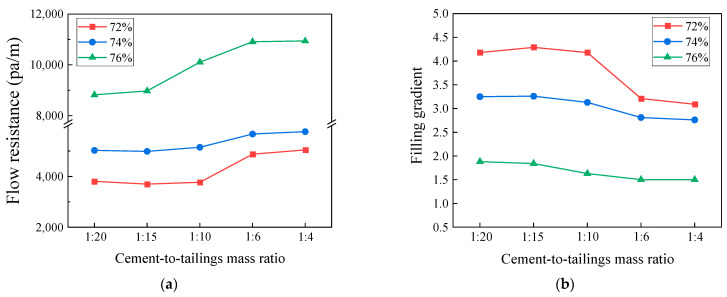
Experiments result for different c/t ratios. (**a**) flow resistance; (**b**) stowing gradient.

The variation in the flow resistance and stowing gradient with the c/t ratio is illustrated in Figure 11. With the growth in the c/t ratio and mass concentration, the transportation resistance of the slurry shows a positive correlation, resulting in a reduction in the flow rate. Consequently, the range of self-flow transportation of the filling slurry becomes narrower, and the stowing gradient also diminishes. In conjunction with Figure 10, as the c/t ratio and mass concentration increase, the slurry yield stress, coefficient of viscosity, and flow resistance clearly show an increase, and the stowing gradient declines. When the c/t ratio rises from 1:20 to 1:4, the composition of solid particles within the slurry varies. The cementitious material BMC accounts for a larger proportion of the slurry, thus providing more raw material for the hydration reactions, allowing the slurry to undergo hydration reactions to a deeper degree, with more hydration products (flocculent C-S-H gels and needle-shaped Aft) uniformly dispersed within the slurry, lapping particles and filling particle voids. Meanwhile, when the mass concentration goes up from 72% to 76%, the free water inside the slurry gets reduced, the solid particles of the tailings increase, the particles contact each other to form a more developed flocculent network structure, and the overall specific surface area becomes larger. Therefore, when the c/t ratio and mass concentration increased, the slurry was more likely to adhere to the pipe wall, leading to a decline in the fluidity of the slurry and a reduction in the flow range. The higher the mass concentration of the filling slurry, the better its viscoplasticity and segregation resistance, and the stronger its mechanical properties after consolidation, but this also leads to an increase in the filling cost. In the goaf, it is indispensable to adjust the slurry preparation parameters by altering the c/t ratio and mass concentration to control the filling slurry to possess an excellent conveying performance, settling performance, and mechanical properties after consolidation. It is essential to arrange a suitable filling network to ensure the successful operation of the filling system and to reduce filling costs by utilizing the self-flowing conveying performance brought about by the height difference of the underground stope.

#### 3.3.3. Semi-Industrial Loop Test Experimental Results

**Figure 12:**
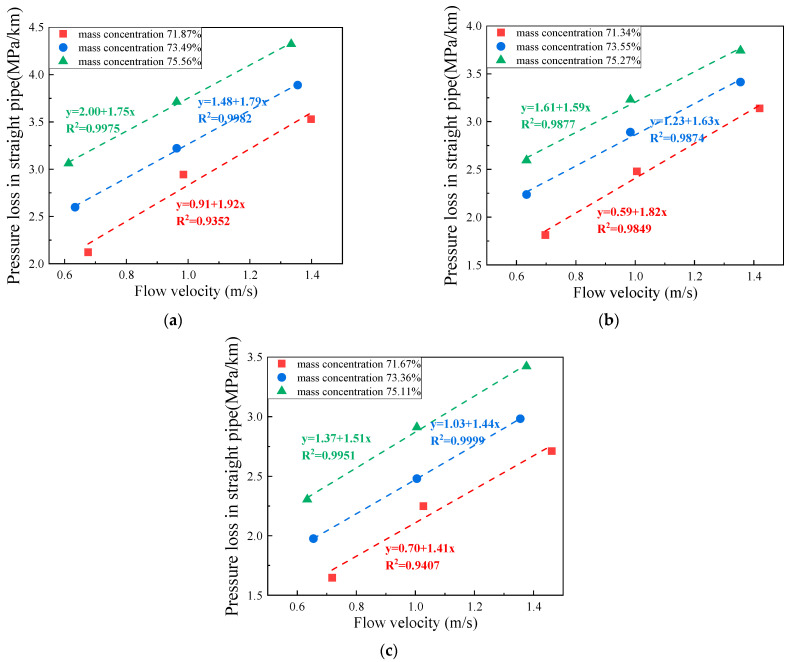
Pressure loss in straight pipe with different c/t ratios: (**a**) 1:4; (**b**) 1:10; and (**c**) 1:20.

Figure 12 demonstrates an approximately linear growth pattern between the pressure loss along the straight pipe section and the slurry flow rate. Under certain conditions of c/t ratio and mass concentration, the pressure loss along the straight pipe section of the CCPB is positively correlated with the flow rate. Taking the filling slurry with a c/t ratio of 1:4 and mass concentration of 76% as an example, when the slurry flow rate is 0.613 m/s, the pressure loss of straight pipe section is 3.061 MPa/km; when the slurry flow rate is 0.963 m/s, the pressure loss of straight pipe section is 3.713 MPa/km; when the slurry flow rate is 1.334 m/s, the pressure loss of straight pipe section is 4.324 MPa/km. As the flow rate increases, the kinetic energy of the CCPB enlarges, and the solid particles move and collide at a faster rate, which intensifies the friction and impact collision between the solid particles inside the slurry and each other and between the solid particles and the pipe wall, increasing the pressure loss in the route. If the flow rate is too high, it will also cause the problem of wall shear. Therefore, if the pressure inside the filling pipe is too high, the pumping capacity can be adjusted to reduce the pumping flow rate to relieve the pressure inside the pipe. In addition, the pressure loss in the straight pipe section tends to grow with an increasing c/t ratio and mass concentration. In nine sets of semi-industrial loop test data, when the slurry c/t ratio increases to 1:4, the mass concentration rises to 76%, and the slurry flow rate reaches the maximum value of 1.334 m/s, while the pressure loss in the route of straight pipe section comes to the maximum value of 4.324 MPa/km. According to the literature [18], the solid particles inside the slurry can be mainly categorized into traction load, suspended load, and jumping load, all of which are affected by the slurry flow rate, c/t ratio, and mass concentration. For a high concentration tailings slurry, when the mass concentration rises from 72% to 74% and then to 76%, the proportion of solid particles, especially tailings in the slurry increases. Consequently, there is more significant mutual collision and friction between the solid particles. Due to its heavy particle weight and easily settled characteristics, the tailings move in the pipeline mainly in the form of a traction load and jumping load, and the friction and impact on the pipeline are relatively large, so the mass concentration is positively correlated with the pipeline pressure drop. In addition to the resistance and wall shear effect, the slurry settlement inside the pipe will lead to a certain pressure loss.

### 3.4. Analysis of Mechanical Properties

#### 3.4.1. Standard Curing Environment

**Figure 13:**
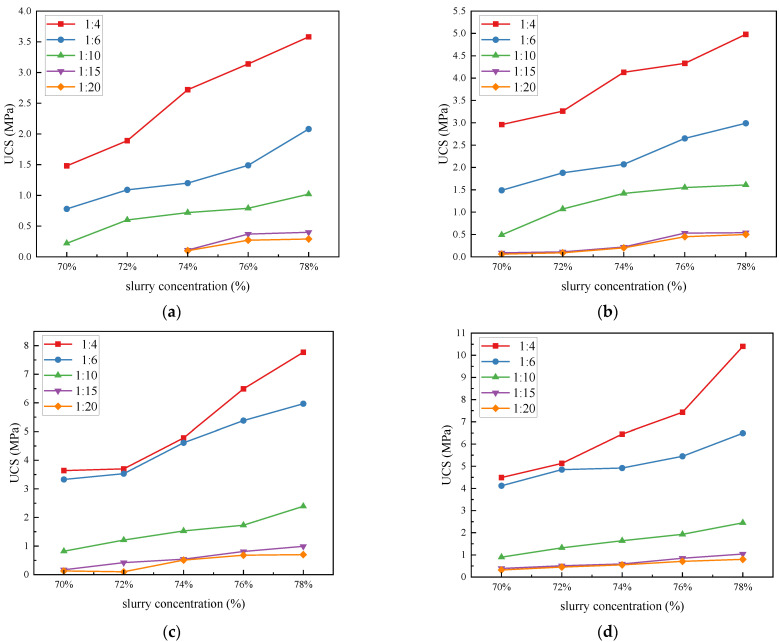
Compressive strength of CCPB in standard laboratory curing environment: (**a**) 3 d; (**b**) 7 d; (**c**) 14 d; and (**d**) 28 d.

**Figure 14:**
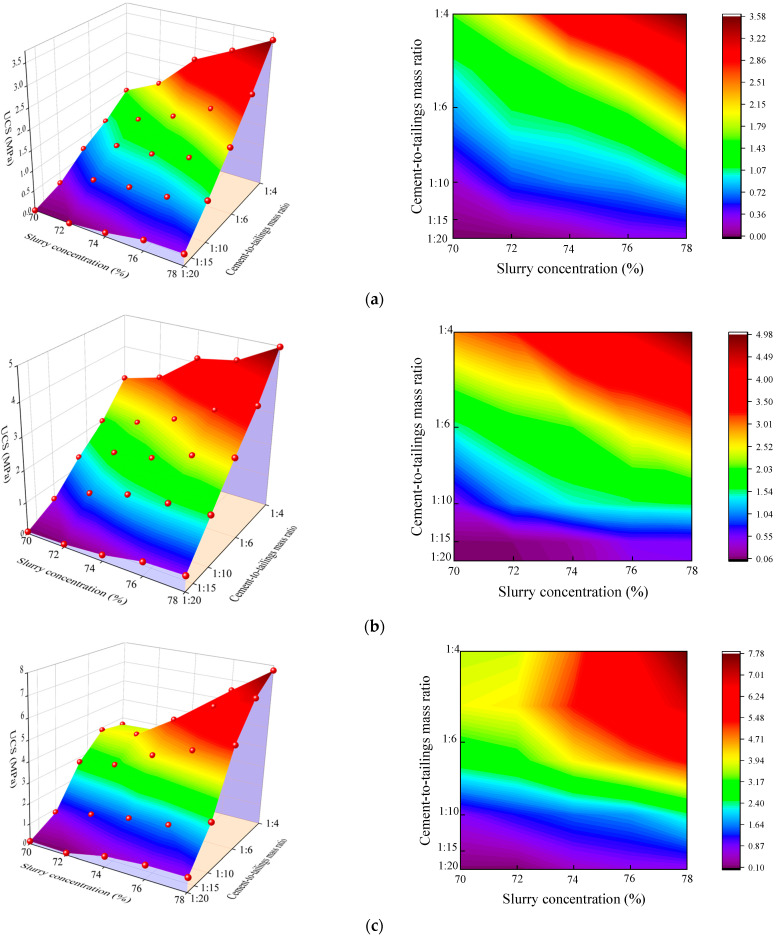

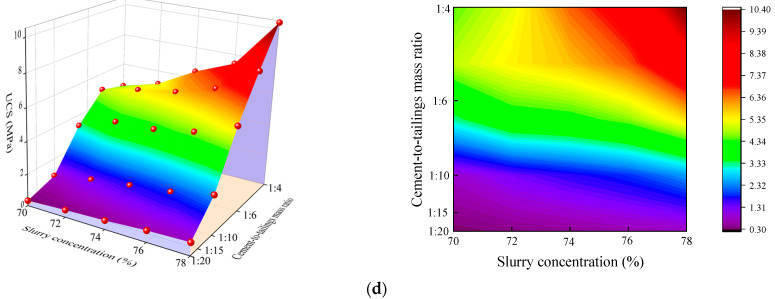
Statistical analysis of compressive strength of CCPB in standard laboratory curing environment: (**a**) 3 d; (**b**) 7 d; (**c**) 14 d; and (**d**) 28 d.

The UCS is an essential reflective marker of the mechanical properties of the filling body. The UCS for different mass concentrations, c/t ratios, and curing ages obtained under standard curing conditions are shown in Figure 13. Figure 14 shows the 3D surface chart and 2D plane projection of the experimental data, which enables us to visualize the effects of slurry mass concentration and c/t ratio on the UCS of the CCPB. The UCS increases with curing age, mass concentration, and c/t ratio, and reaches the maximum value of 10.4% when the age is 28 d, the mass concentration is 78%, and the c/t ratio is 1:4. Under a certain c/t ratio, the mass concentration of whole tailings filling slurry has a direct relationship with the UCS. Taking the specimen with a c/t ratio of 1:4 and curing age of 28 d as an example, with an increase in the slurry mass concentration from 70% to 78%, the UCS increases from 4.49 MPa to 5.13 MPa, 6.45 MPa, 7.43 MPa, and 10.4 MPa with the increasing range of 14.25%, 43.6%, 65.48%, and 131.63%, respectively. The higher the mass concentration, the more BMC and other solid particles occupying the slurry, the greater the strength of the CCPB, and the more supportive of the goaf, so the mass concentration of the filling slurry should be enhanced as much as possible. Under the condition of a certain mass concentration of the filling slurry, the added amount of cementitious material directly affects the strength of the filling body. When the consumption of cementitious materials ascends, the raw materials for the hydration reaction rise, which contributes to the hydration reaction, generating more hydration products and further enhancing the strength of the CCPB. From the curve slope, it can be judged that when the mass concentration gradually goes up from 70% to 78%, the UCS of the high c/t ratio (1:6 and 1:4) specimens is found to increase sharply faster than that of the low c/t ratio specimens (1:15 and 1:20). When the slurry c/t ratio is too low, the slurry is prone to segregation, delamination, and bleeding, which harms the overall strength of the CCPB. The strength of the filling body leaps with an increasing curing age. Figure 13a,b demonstrate that in the early stage of hydration reaction, the UCS of the CCPB with a low c/t ratio (1:15 and 1:20) is extremely weak. The UCS of the CCPB with a high c/t ratio (1:4) is considerably stronger than that of the other filling bodies. Figure 13c,d illustrate that the CCPB strengths of low c/t ratios (1:10, 1:15, and 1:20) do not exceed 2 MPa with a long curing age. The CCPB strengths of high c/t ratios (1:6 and 1:4) are comparatively similar. As the slurry mass concentration sustainably grows, the UCS of the CCPB with a c/t ratio of 1:4 is stronger. Taking the specimen with a c/t ratio of 1:4 and mass concentration of 78% as an example, its peak strength drastically increases by 190.50% as the curing age rises from 3 d to 28 d. This is attributed to the hydration reaction inside the specimen steadily tending to be mature, and the hydration products represented by the C-S-H gel and AFt fill the pores and lap the tailings particles, which reduces the porosity of the CCPB, enhances its cohesive forces, and improves the overall structural density to a large extent.

#### 3.4.2. Deep-Underground Curing Environment

**Figure 15:**
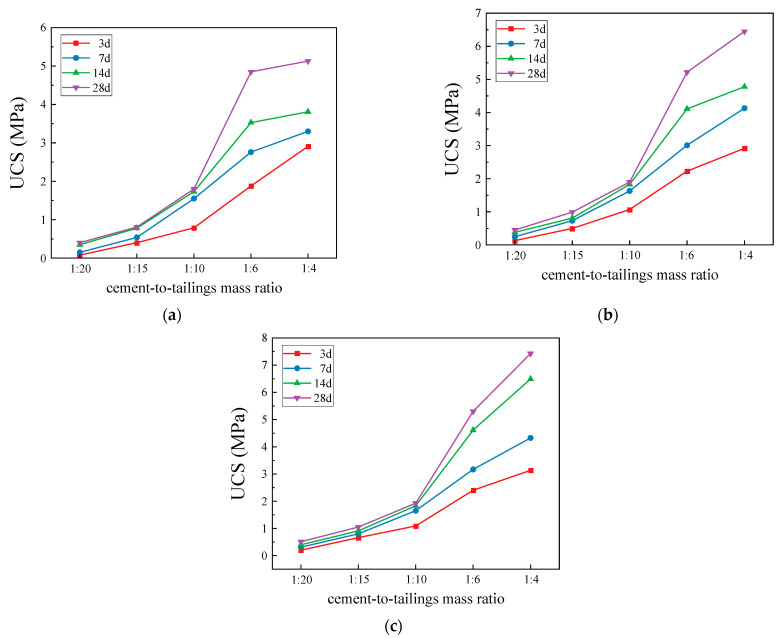
Compressive strength of CCPB in deep-underground curing environment: (**a**) 72%; (**b**) 74%; and (**c**) 76%.

**Figure 16:**
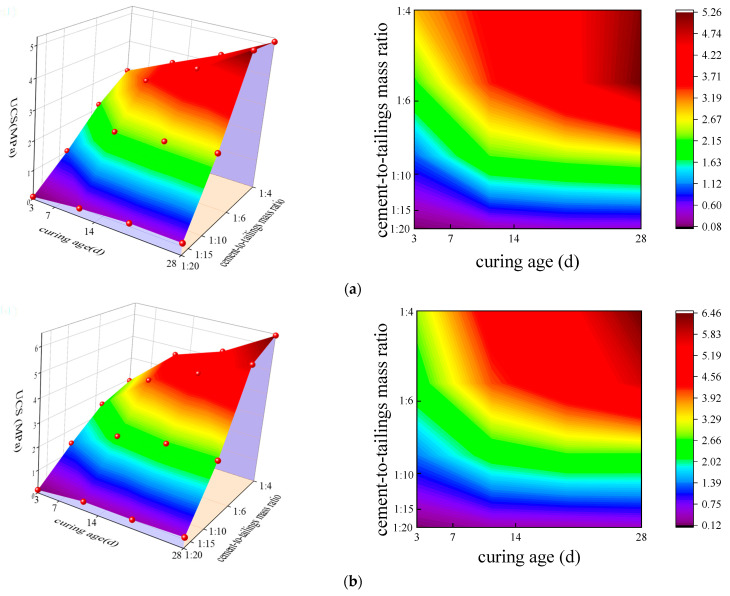

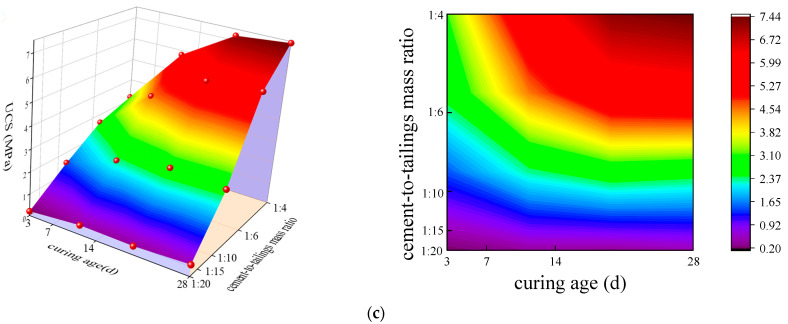
Statistical analysis of compressive strength of CCPB in deep-underground curing environment: (**a**) 72%; (**b**) 74%; and (**c**) 76%.

Figure 15 shows the results of the UCSs obtained from the deep-underground curing environment with different mass concentrations, c/t ratios, and curing ages. Figure 16 presents the uniaxial experimental data’s 3D surface chart and 2D plane projection. Compared with the standard curing environment, the UCS of the CCPB in the deep-underground curing environment shows the same increasing trend, i.e., the UCS is positively correlated with the curing age, c/t ratio, and mass concentration. The slope of the curve reaches its peak when the c/t ratio increases from 1:10 to 1:6, and the growing trend appears to be more remarkable. Additionally, when the curing age goes above 7 d, the UCS of the CCPB can reach the state of high stress after the c/t ratio exceeds 1:6. This indicates that to achieve a high filling strength, the c/t ratio should not be lower than 1:6 in the practical production of this mine. As the slurry c/t ratio continuously grows to 1:4, the strength of CCPB reaches its maximum value, which is 5.13 MPa, 6.45 MPa, and 7.43 MPa at different mass concentrations with a curing age of 28 d.

As seen from the 2D plane projection of Figure 16, contrasting with the standard curing environment, the deep-underground curing environment with a curing temperature of 30 °C and a humidity of 90% exhibits a larger area occupied by high stress. This effect is particularly notable when the c/t ratio is increased from 1:6 to 1:4, which demonstrates that the utilization of BMC as a cementitious material in the filling slurry can be better applied to the deep-underground environment of this mine. Combined with the results of the slurry conveying experimental research and the comprehensive judgment of the mine’s economic benefits, when the whole tailings filling slurry is at c/t ratio of 1:4 and mass concentration of 74% or so, the slump, extension degree, bleeding rate, flow properties, and other indexes of the filling slurry satisfy the transportation and filling requirements, the filling body possesses a high strength and can well support the goaf.

## 4. Conclusions

Based on laboratory experiments in the standard curing environment and the deep-underground curing environment, this research analyzes the effects of different c/t ratios and mass concentrations on the performance of the CCPB in terms of the filling slurry flow properties and mechanical performance of the filling body. As a result, the following conclusions are drawn:

(1) When the c/t ratio is 1:4, the filling slurry with a mass concentration between 74% and 76% can be regarded as a paste, suitable for pipeline transportation in the mining industry.
(2) The transportation resistance of the slurry is positively correlated with the c/t ratio and mass concentration, and as the flow rate declines, the range that can be reached by self-flow transportation of the filling slurry becomes narrower, and the stowing gradient also decreases.
(3) On the one hand, the pressure loss in the route of the straight pipe section shows an approximately linear growth pattern with the slurry flow rate. On the other hand, the pressure loss in the straight pipe section tends to grow with an increasing c/t ratio and mass concentration. In nine sets of semi-industrial loop test data, when the slurry c/t ratio increases to 1:4, the mass concentration rises to 76%, and the slurry flow rate reaches the maximum value of 1.334 m/s, the pressure loss in the route of the straight pipe section reaches the maximum value of 4.324 MPa/km.
(4) The UCS of the CCPB in both the standard curing environment and the deep-underground curing environment improved with an increasing curing age, mass concentration, and c/t ratio. The UCS of the CCTB cured in a deep-underground curing environment is relatively stronger and possesses higher stresses when the c/t ratio is between intervals of 1:6 and 1:4. The filling slurry using BMC as the cementitious material can be better applied to the deep-underground environment of this mine.
(5) Combined with the results of the slurry conveying experimental research and the comprehensive judgment of the mine’s economic benefits, when the whole tailings filling slurry is at c/t ratio of 1:4 and mass concentration of 74% or so, the slump, extension degree, bleeding rate, flow properties, and other indexes of the filling slurry satisfy the transportation and filling requirements, the filling body possesses a high strength and can well support the goaf.

## Figures and Tables

**Table 1:** Physical parameters of tailings.

Physical Parameters	True Density (g/cm^3^)	Bulk Density (g/cm^3^)		Percentage of Void (%)		Natural Repose Angle (°)
		Loose	Compacted	Loose	Compacted	
The properties of tailings	2.65	1.15	1.54	56.60	41.89	41.11

**Table 2:** Physical parameters of BMC.

Physical Parameters	True Density (g/cm^3^)	Bulk Density (g/cm^3^)	Percentage of Void (%)	Natural Repose Angle (°)	Specific Surface Area (m^2^/kg)	Initial Consolidation Time (h)	Final Consolidation Time (h)
BMC properties	2.80	1.02	61.94	26.21	750	6	9

**Table 3:** Results of L-tube self-flow test.

C/t Ratio	Mass Concentration (%)	Bulk Density (N/m^3^)	Flow Speed (m/s)	Shear Stress on Tube Wall (pa)	Rheological Parameters		Flow Resistance (pa/m)	Stowing Gradient
					Yield Shear Stress (pa)	Coefficient of Viscosity (pa·s)		
1:4	72	18,222	2.78	126.09	47.86	0.28	5043.41	3.09
	74	18,648	0.85	144.15	92.30	0.31	5765.87	2.76
	76	19,094	0.30	273.56	198.87	0.35	10,942.40	1.50
1:6	72	18,194	2.77	121.91	41.57	0.30	4876.27	3.21
	74	18,618	0.86	141.83	89.86	0.32	5673.17	2.81
	76	19,062	0.42	272.76	195.50	0.36	10,910.51	1.50
1:10	72	18,169	2.05	94.28	26.43	0.36	3771.20	4.18
	74	18,591	0.80	128.73	78.79	0.37	5149.33	3.13
	76	19,033	0.39	252.67	177.80	0.50	10,106.67	1.63
1:15	72	18,156	1.98	92.47	25.40	0.37	3698.99	4.29
	74	18,577	0.76	124.70	76.20	0.38	4988.16	3.26
	76	19,017	0.33	224.29	157.92	0.52	8971.52	1.84
1:20	72	18,149	1.99	95.15	24.80	0.39	3806.19	4.18
	74	18,569	0.78	125.72	75.10	0.41	5028.69	3.25
	76	19,009	0.35	220.48	155.70	0.46	8819.20	1.88

**Table 4:** Pipe pressure loss results of slurry with c/t ratio of 1:4.

Design Mass Concentration (%)	Observe Mass Concentration (%)	Pumping Frequency (/min)	Flow Velocity (m/s)	Pressure Loss (MPa/km)				
				Straight Pipe	30° Tilt Downward	30° Tilt Upward	Vertically Downward	Vertically Upward
72	71.87	14	0.676	2.126	0.611	0.611	0.443	20.138
		15	0.984	2.947	0.683	0.683	0.580	21.268
		21	1.397	3.531	1.042	1.042	0.626	22.061
74	73.49	12	0.634	2.598	0.653	0.653	0.530	21.156
		14	0.963	3.221	0.817	0.817	0.691	22.056
		19	1.355	3.890	1.246	1.246	0.776	22.662
76	75.56	11	0.613	3.061	0.838	0.838	0.631	21.934
		14	0.963	3.713	1.076	1.076	0.743	22.515
		18	1.334	4.324	1.321	1.321	0.902	23.370

## Data Availability

Data are contained within the article.
